# A Neurochemical Basis for Phenotypic Differentiation in Alzheimer's Disease? Turing's Morphogens Revisited

**DOI:** 10.3389/fnagi.2017.00076

**Published:** 2017-03-29

**Authors:** Heather T. Whittaker, Jason D. Warren

**Affiliations:** ^1^Department of Neurodegenerative Disease, UCL Institute of Neurology, University College LondonLondon, UK; ^2^Dementia Research Centre, UCL Institute of Neurology, University College LondonLondon, UK

**Keywords:** Alzheimer's disease, dementia, phenotype, network, Turing, protein

## Introduction

The pathological process underpinning Alzheimer's disease (AD) can manifest as any of several distinctive clinico-anatomical syndromes (Warren et al., 2012). The factors that drive this phenotypic variation remain unclear but are likely to hold important insights into the mechanisms whereby local neurotoxic effects of pathogenic proteins are scaled to distributed brain networks. A theoretical framework for understanding morphological differentiation in biological systems was first outlined in now classic work by Turing (1952), who showed computationally that diffusion of two or more tissue chemicals or “morphogens” reacting across an embryonic cellular network is sufficient to scale initial random fluctuations into stable, often strikingly asymmetric patterns. Here we propose that Turing's theory predicts the phenotypic diversity of AD, as a fundamental consequence of two interacting pathogenic proteins (phosphorylated tau and beta-amyloid) that spread diffusively through a common, distributed neural network.

## The problem of alzheimer's disease: clinical diversity on a common pathological substrate

Canonically, AD presents with episodic memory impairment attributable to dysfunction of hippocampi and connected circuitry traversing the mesial temporal lobes. However, several other variant clinical presentations of AD are well-recognized: these include a “visual” variant led by visuoperceptual and visuospatial deficits; “logopenic aphasia” led by language impairment; and a “frontal” variant led by executive and behavioral decline (Warren et al., 2012). These clinical syndromes have associated profiles of regional brain dysfunction and atrophy which are likely to reflect differential involvement of a core, distributed temporo-cingulo-parietal network and its projections by the diffusive spread of pathogenic proteins (Seeley et al., 2009; Pievani et al., 2011; Warren et al., 2012). In the healthy brain, the core network mediates stimulus-independent thought (hence its designation as the so-called “default-mode network”) and it is targeted early and relatively selectively by the pathological process in AD (Buckner et al., 2009; Simic et al., 2014).

All AD phenotypes are characterized by pathological tissue accumulation of neurofibrillary tangles containing abnormally-phosphorylated tau and extracellular plaques containing beta-amyloid. Though their precise relation remains contentious, beta-amyloid and phosphorylated tau are central to current concepts of AD biology, and the action of toxic oligomers on synapses may instigate a cascade of intra- and extra-cellular events leading ultimately to the tissue expression of AD (Ittner and Götz, 2011; Morris et al., 2014). While the regional tissue distribution of pathology may vary between AD syndromes (Murray et al., 2011; Mesulam et al., 2014; Martersteck et al., 2016; Ossenkoppele et al., 2016), any mapping between histology and phenotype is likely to be complex. This raises an apparent paradox: why should the pathological process in AD manifest as a handful of diverse but consistent patterns, rather than as a single uniform signature or a stochastic spectrum of random tissue damage?

## Turing's theory of morphogenesis and its legacy

Turing's reaction–diffusion theory of morphogenesis posits that an initially stable, homogeneous cell array containing “form-producing” chemicals (morphogens) may depart from stability due to stochastic fluctuations in the array. It is assumed that morphogens diffuse and react, such that a morphogen may excite its own formation and diffusion by autocatalysis, or inhibit these processes in another morphogen; it is further required that morphogens have different rates of diffusion. Excitation-inhibition coupling between the morphogens tends to focus autocatalysis locally into zones separated by intervening regions where inhibitory effects predominate (if this is not the case, then catastrophic instability occurs and growth of the reaction will halt). Over time, a “wave-like” pattern of inhomogeneous morphogen concentrations develops across the cell array and transmits a corresponding pattern of cellular effects. For the case of two interacting morphogens, the resultant patterns resemble standing waves and become more salient over time, while for three or more morphogens, more complex behaviors emerge.

Turing showed that it is relatively straightforward mathematically to extend the reaction–diffusion framework of “homogeneity breakdown” from a ring to a sphere (or shell) of cells. Since Turing's original formulation, his theory has been shown to hold for an extraordinary variety of applications, ranging from coat pigmentation patterns in animals to predator-prey relationships in ecosystems, crime hotspots in communities, sand ripples, and galaxy formation (Murray, 1990; Ball, 2015). Turing effects have also been shown to operate on electrophysiological neural network parameters that do not require physical transfer of “morphogens” (Jirsa and Kelso, 2000; Hutt and Atay, 2005; Steyn-Ross et al., 2009, 2013).

## Translating turing: from morphogens to neurodegenerative pathogens

**Figure 1:**
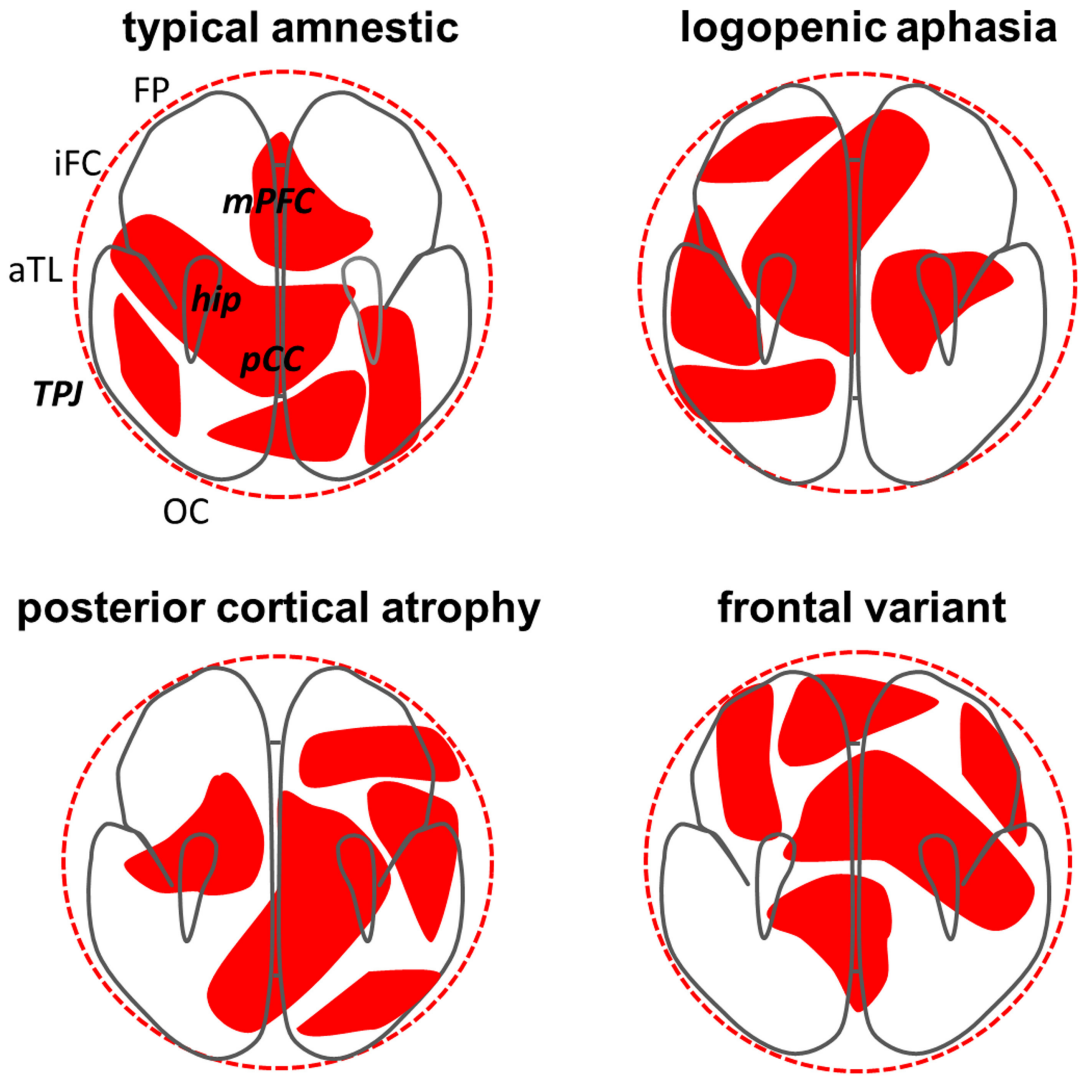
**Phenotypic differentiation in Alzheimer's disease interpreted as a Turing reaction—diffusion process**. Each panel shows a schematic axial projection of the cerebral hemispheres and the top left panel indicates key anatomical regions, including components of the “default-mode network” (in bold italics): aTL, anterior temporal lobe; FP, frontal pole; hip, hippocampus; iFC, inferior frontal cortex; mPFC, medial prefrontal cortex; TPJ, temporo-parietal junction; OC, occipital cortex; pCC, posterior cingulate cortex. Superimposed (in red) on the brain schemas in each panel are notional patterns of pathogenic protein (phosphorylated tau and beta-amyloid) effects resulting from a Turing mechanism, each operating for the same arbitrary (clinically relevant) time period; these patterns correspond broadly to the atrophy profiles observed in the designated major variant syndromes of Alzheimer's disease (see Warren et al., 2012). The ideal “spherical” diffusion volume (dotted outer circle) is constrained by the geometry of brain boundaries; together these factors govern the number of possible patterns that can develop and their effective “wavelength.” Targeting of the default-mode network by Alzheimer pathology may reflect intrinsic vulnerability of this network to Turing effects (Steyn-Ross et al., 2009, 2013). The patterns in each panel here are related (purely for illustrative purposes) as simple rotations of a single template pattern that preserve involvement of the key default-mode network but alter the profile of involvement across network components, each profile corresponding to a major Alzheimer phenotype. Degree of “rotation” in this context could signify chemotactic and mechanical factors that modulate expression of the basic reaction–diffusion process (Murray, 1990; Ball, 2015; Kondo, 2016) or variation in specific network connectivity parameters (Jirsa and Kelso, 2000; Hutt and Atay, 2005; Steyn-Ross et al., 2009, 2013). This simplified model illustrates several cardinal features of Alzheimer phenotypes: (i) network “hubs” such as posterior cingulate and hippocampus are involved in each case; (ii) there is substantial overlap between rotated patterns (syndromic variants); (iii) at the same time, each variant involves additional, connected, syndrome-specific brain regions beyond the core network. We propose that other (non-Alzheimer) neurodegenerative proteinopathies may have analogous but disease-specific “Turing signatures.”

Our proposal to extend Turing's theory to AD pathogenesis was motivated by the empirical resemblance of AD neuroanatomical phenotypes to Turing reaction-diffusion patterns in other biological and physical systems. The two pathogenic proteins integral to the development of AD are clearly dissimilar to the morphogens of developmental biology (Tiberi et al., 2012): the pathogenic proteins of AD are “form-destroyers” rather than form-producers and any analogy must be qualified. Nevertheless, these AD proteins are likely to possess the key Turing morphogen attributes of diffusive spread and mutual reaction (Ittner and Götz, 2011; Warren et al., 2013): the “inhomogeneities” they produce are departures from brain network health, expressed as regional neural dysfunction and damage. Our idea is sketched in Figure 1.

Pathogenic protein effects on synaptic function and inter-cellular connectivity determine the final common pathway of protein diffusion and reaction at network level. Computational modeling of neural network behavior has established that certain synaptic connectivity properties can generate spatial Turing instabilities over macroscopic scales via long-range electrophysiological field effects (Jirsa and Kelso, 2000; Hutt and Atay, 2005; Steyn-Ross et al., 2009, 2013). In particular, Turing activation patterns emerge where the firing rates of connected neurons are governed by disproportionate excitatory vs. inhibitory inputs acting over different spatial ranges. Besides physical diffusion between neurons (Warren et al., 2013), tau and beta-amyloid have complex effects on synaptic and neurotransmitter physiology that might establish such Turing field effects. These proteins react extensively in an intricate “pas de deux” that is likely to produce net toxic gain-of-function as well as loss-of-function effects at synaptic (and by extension, network) level (Winklhofer et al., 2008; Ittner and Götz, 2011; Leighton and Allison, 2016; Ovsepian et al., 2016). While ultimately the interaction of tau and beta-amyloid is additive in promoting the spread of AD pathology, at a given stage during evolution of the disease the proteins might plausibly have mutually reciprocal effects on synaptic function and network connectivity: for example, prior to undergoing pathogenic misfolding tau protein protects against beta-amyloid-induced neuronal dysfunction (Dawson et al., 2010), while tau and beta-amyloid associate with distinct network profiles in the aging brain (Sepulcre et al., 2016).

Remarkably, connectivity properties of the default-mode network may make it intrinsically more susceptible to Turing effects than other large-scale brain networks (Steyn-Ross et al., 2009, 2013). This might explain why the network is selectively targeted by the dual-protein pathological process in AD and why this process is phenotypically differentiated. Electrophysiological Turing patterns of neuronal dysfunction developing within the network would be translated into neuronal damage and death, thereby fixing the electrophysiological patterns into the structural atrophy patterns that constitute AD phenotypes (Figure 1).

## Translating turing: some key problems

We now consider certain important challenges in extending the Turing framework to AD.

One immediate consideration is the geometry (and relatedly, the finite number) of diffusive AD patterns. Turing structures are highly dependent on system boundary and scaling constraints as well as specific diffusion characteristics of the relevant morphogens, which are generally not known a priori (Murray, 1990). The intrinsic “wavelength” of the putative reaction–diffusion process in AD is likely to be substantially larger than an individual cell or cortical column and may be amplified by involvement of longer-range network projections, which have been shown to support macroanatomical Turing structures (Nakamasu et al., 2009; Steyn-Ross et al., 2009, 2013; Kondo, 2016).

While Turing's concept of autocatalysis is broadly supported by empirical evidence for auto-propagation of pathogenic proteins in AD and other proteinopathies (Hardy and Revesz, 2012; Warren et al., 2013), the relation between phosphorylated tau and beta-amyloid remains a key unresolved issue. A Turing model would require them to fill the roles of “activator” and “inhibitor:” this need not of course imply that either protein has a protective role, but would predict a reciprocal relationship over some spatial scale or temporal interval. At present this is difficult to assess directly in human disease, however it may be pertinent that phenotype and tissue damage have been found generally to correlate with the regional distribution of phosphorylated tau but not beta-amyloid (Morris et al., 2014; Ossenkoppele et al., 2016).

A further key issue is that the clinico-anatomical patterns constituting AD phenotypes are eventually unstable, evolving and converging to a global distribution of tissue damage with disintegration of the network that instantiates the reaction–diffusion process. As originally proposed, Turing's theory eschewed scenarios far from the onset of inhomogeneity and did not explicitly model changing temporal dynamics: these scenarios are clearly apposite to AD and may be more effectively addressed using more recent extensions of the theory, including the incorporation of temporal Hopf instabilities (Kondo et al., 2009; Steyn-Ross et al., 2009, 2013; Kondo, 2016).

## Testing the idea and future directions

In its simplest form, Turing's model depends on four parameters for each pathogenic protein: the rate of production; the rate of diffusion; the rate of degradation; and the magnitude of their interaction. This should make the model relatively amenable to experimental evaluation in artificial neural networks using computational techniques or indeed, *in vitro* neural circuits or transgenic animals. Computational models incorporating biologically-realistic neuronal and circuit parameters have been shown to generate complex Turing behavior for both the healthy brain and selected disease states such as epilepsy and schizophrenia (Jirsa and Kelso, 2000; Steyn-Ross et al., 2009, 2013). These models should be extended to simulate the effects of pathogenic protein properties on synaptic function and tissue spread. The advent of tau-PET neuroimaging (in conjunction with well-established amyloid imaging) opens an avenue to directly compare tau and beta-amyloid tissue deposition profiles in patients (Ossenkoppele et al., 2016). Ultimately there is a need for direct histopathological examination of human brain tissue: while inevitably subject to ascertainment bias (toward more advanced disease), this could be somewhat offset by improved definition of the culprit molecular species and their sites of action within local tissue circuits (Ittner and Götz, 2011; Morris et al., 2014). Although it is unlikely that a Turing reaction-diffusion process is the sole influence governing phenotypic differentiation in AD, it might act as an essential driver that is modulated by other endogenous and environmental factors (Murray, 1990; Ball, 2015; Kondo, 2016).

The molecular nexopathies paradigm of neurodegeneration rests on a coherent conjunction of pathogenic protein and network characteristics (Warren et al., 2013). Turing effects might underpin the peculiar vulnerability of the brain's default-mode network to AD nexopathy (Steyn-Ross et al., 2009). At the same time, a Turing model of AD pathogenesis might suggest that the neurodegenerative process “unravels” the events of normal neural network ontogeny (Tiberi et al., 2012), implying that embryological differentiation and disease-related de-differentiation exploit intrinsically similar mechanisms. An important motivation for examining models such as Turing's in this context is to deconstruct the apparent complexity of neurodegenerative disease phenomenology to more tractable building blocks. Phenotypic heterogeneity in AD is often ascribed to the operation of still unidentified genetic and epigenetic modifiers of disease expression in particular neural systems (Murray et al., 2011; Warren et al., 2012; Mesulam et al., 2014; Martersteck et al., 2016; Ossenkoppele et al., 2016): if valid, a Turing process would provide a parsimonious mechanism encompassing all variant phenotypes and inherent to the primary disease. This in turn might have implications for development of novel biomarkers and therapeutic interventions targeting the factors that scale reaction–diffusion processes dynamically across the compromised network. At least in principle, macroanatomical confirmation of a Turing signature could help discriminate between candidate molecular mechanisms that drive the observed patterns of neural damage (Kondo et al., 2009; Kondo, 2016).

Finally, Turing's theory may be broadly applicable to other forms of pathological aging and a range of neurodegenerative proteinopathies besides AD. The interaction of C9orf72 products and TDP-43 in frontotemporal dementia is one recent candidate (Vatsavayai et al., 2016) but the application need not be restricted to diseases with two protein pathogens; the role of the second morphogen in the Turing model might be taken by a non-pathogenic tissue factor. Human neurodegenerative diseases may further vindicate the far-reaching potency of Turing's original idea.

## Author contributions

Both HW and JW contributed substantially to the conception of the work, drafted, and revised the work critically for important intellectual content and gave final approval of the version to be published. Both agree to be accountable for all aspects of the work in ensuring that questions related to the accuracy or integrity of any part of the work are appropriately investigated and resolved.

### Conflict of interest statement

The authors declare that the research was conducted in the absence of any commercial or financial relationships that could be construed as a potential conflict of interest.
